# Characterization of the stem cell system of the acoel *Isodiametra pulchra*

**DOI:** 10.1186/1471-213X-9-69

**Published:** 2009-12-18

**Authors:** Katrien De Mulder, Georg Kuales, Daniela Pfister, Maxime Willems, Bernhard Egger, Willi Salvenmoser, Marlene Thaler, Anne-Kathrin Gorny, Martina Hrouda, Gaëtan Borgonie, Peter Ladurner

**Affiliations:** 1 University of Innsbruck, Institute of Zoology, Technikerstrasse 25, A-6020 Innsbruck, Austria; 2 University of Ghent, Department of Biology, Ledeganckstraat 35, B-9000 Ghent, Belgium; 3 Carl Zeiss NTS GmbH, Carl-Zeiss Str 56, D-73447 Oberkochen, Germany; 4 Department of Biophysics, Graduate School of Science, Kyoto University, Kitashirakawa-Oiwake, Sakyo-ku, Kyoto 606-8502, Japan; 5 Current address: Hubrecht Institute, Uppsalalaan 8, 3584 CT Utrecht, The Netherlands

## Abstract

**Background:**

Tissue plasticity and a substantial regeneration capacity based on stem cells are the hallmark of several invertebrate groups such as sponges, cnidarians and Platyhelminthes. Traditionally, Acoela were seen as an early branching clade within the Platyhelminthes, but became recently positioned at the base of the Bilateria. However, little is known on how the stem cell system in this new phylum is organized. In this study, we wanted to examine if Acoela possess a neoblast-like stem cell system that is responsible for development, growth, homeostasis and regeneration.

**Results:**

We established enduring laboratory cultures of the acoel *Isodiametra pulchra *(Acoela, Acoelomorpha) and implemented *in situ *hybridization and RNA interference (RNAi) for this species. We used BrdU labelling, morphology, ultrastructure and molecular tools to illuminate the morphology, distribution and plasticity of acoel stem cells under different developmental conditions. We demonstrate that neoblasts are the only proliferating cells which are solely mesodermally located within the organism. By means of *in situ *hybridisation and protein localisation we could demonstrate that the *piwi*-like gene *ipiwi1 *is expressed in testes, ovaries as well as in a subpopulation of somatic stem cells. In addition, we show that germ cell progenitors are present in freshly hatched worms, suggesting an embryonic formation of the germline. We identified a potent stem cell system that is responsible for development, homeostasis, regeneration and regrowth upon starvation.

**Conclusions:**

We introduce the acoel *Isodiametra pulchra *as potential new model organism, suitable to address developmental questions in this understudied phylum. We show that neoblasts in *I. pulchra *are crucial for tissue homeostasis, development and regeneration. Notably, epidermal cells were found to be renewed exclusively from parenchymally located stem cells, a situation known only from rhabditophoran flatworms so far. For further comparison, it will be important to analyse the stem cell systems of other key-positioned understudied taxa.

## Background

The question how adult organisms maintain their tissue homeostasis or perform wound healing and regeneration after injury touches different biological and medical research areas. The two main invertebrate model organisms, *Drosophila melanogaster *and *Caenorhabditis elegans *are largely post-mitotic and therefore cannot serve as model systems for tissue renewal nor for the biology of somatic stem cells. Vertebrate stem cell systems have been addressed because of their medical relevance, but the accessibility of these stem cell systems is limited. Flatworms are well known for their remarkable totipotent stem cell system. These stem cells (so called neoblasts) are the sole source for cell renewal during homeostasis, development and regeneration [1-8], and give rise to all cell types including germ cells [9,10]. A basal member of the Platyhelminthes - the Acoela - became separated from other flatworms [11-20] by molecular phylogeny and were placed as a sistergroup to all Bilateria [16,19,20], associated with the Deuterostomes [17] or located within the Lophotrochozoa [18]. Already 20 years ago, the question whether acoel flatworms are "Kingpins of Metazoan evolution or specialized offshoot" [21] has been raised by summarizing data of a century of morphological analyses where Acoelomorpha have been associated to the phylum Platyhelminthes [22]. By contrast, recent data on the distribution and proliferation of stem cells and the specific mode of epidermal replacement could constitute for a possible synapomorphy between the Acoela and the major group of flatworms, the Rhabditophora [19]. Like rhabditophoran flatworms, certain acoels exhibit tremendous capacity to regenerate lost body parts [23,24] or show modes of asexual reproduction such as reverse-polarity budding [25,26].

Despite the growing interest in acoel phylogeny, knowledge on the developmental biology of this taxon is limited. Few reports described the embryonic muscle development [27], the characteristic spiral duet cleavage [28], while others examined their stem cell system and showed that acoels possess also neoblasts which resemble stem cells of rhabditophoran flatworms [19,29,30]. However, very little is known on the cellular and molecular basis that is driving homeostasis, asexual reproduction and regeneration in these organisms. Research on acoels has been hampered by the availability of an acoel species that can be cultured and used as a suitable model system. Here we present the acoel *Isodiametra pulchra *(Acoela, Acoelomorpha) as an adequate species to address developmental and evolutionary questions. *I. pulchra *has several advantages to perform these analyses: (1) long term laboratory cultures can be maintained, (2) the animals are small in size (1 mm), (3) reproduce rapidly (one egg per animal per day the whole year through), (4) have a very short embryonic development (36 hours) [27], (5) a short generation time (one month), (6) 14.000 ESTs have been sequenced (Ladurner and Agata, unpubl.), and (7) *in situ *hybridization and RNA interference protocols are established (see below).

The last decennia, the stem cell system of flatworms has been characterized on a molecular level [31-35]. Some of the well characterized stem cell regulatory genes in flatworms belong to the *piwi-*like gene family [33,34,36,37]. In most organisms studied so far, PIWI is a germline specific marker, essential in spermatogenesis, meiosis and germ cell maintenance where it is involved in transposon regulation [38-42]. An exception herein are rhabditophoran flatworms, sponges and cnidarians where *piwi*-like genes have been shown to play an extended role in somatic stem cells [33,36,37,43-45].

Here we show that in *I. pulchra, piwi *is also expressed in a subpopulation of somatic neoblasts. Next, we report on the morphology of stem cells, their distribution and differentiation capacity in this acoel species. Furthermore, we studied the function of the stem cell system during homeostasis, development, regeneration, hydroxyurea treatment, starvation and after irradiation using histology, electron microscopy, BrdU labelling, *in situ *hybridization and RNA interference. To summarize, these data provide new insides how stem cell systems might have been developed during animal evolution.

## Results

### Morphology, distribution, and differentiation of stem cells in *Isodiametra pulchra*

**Figure 1:**
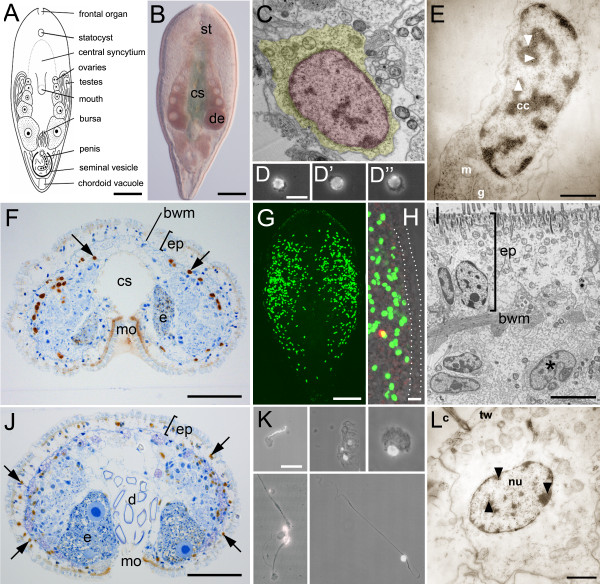
**The stem cell system of *Isodiametra pulchra *(A, B). Morphology (C-E), distribution (F-I), and differentiation (J-L) of neoblasts**. (A) Schematic drawing. (B) Differential interference contrast image. (C) Typical neoblast with nucleus (red) and thin rim of cytoplasm (yellow). (D-D") Macerated BrdU labelled cells show typical neoblast like morphology (E) BrdU labelled neoblast, as shown by immunogold staining after a 30 min BrdU pulse; arrowheads point to gold particles (F) histological cross section; brown spots are BrdU labelled S-phase cells. (G, H) Confocal projection overview (G) and detail of lateral body margin (H) after 30 min BrdU pulse; the red spot in (H) is a mitotic figure. Note that S-phase cells were lacking in the epidermis (between dotted lines). (I) Electron microscopic image of a posterio-lateral body margin. (J) Histological section, 10 days after the initial BrdU pulse. Some of the neoblasts underwent differentiation into epidermal cells (arrows); (K) BrdU labelled cells, differentiated after 10 days chasing time. Differentiating spermatid (top left), epidermal cells (top middle), parenchymal cell (top right), nerve cells (bottom left), and a muscle cell (bottom right) (L) BrdU labelled differentiated epidermal cell after 10 days chasing; arrowheads point to gold particles. bwm, body wall musculature; c, cilium; cc, condensed chromatin; cs, central syncytium; d, diatoms; e, egg; de, developing eggs; ep, epidermis; g, golgi; m mitochondria; mo, mouth opening; n nucleolus; st, statocyst; tw, terminal web. Scale bars (A, B, G) 100 μm; (C, E, L) 1 μm; (D, H; K) 10 μm; (F, J) 25 μm; (I) 5 μm.

In order to describe the stem cell system of acoels, we first addressed the morphology of *Isodiametra pulchra *(Figures. 1A, B) neoblasts. They are small in size and possess a high nuclear to cytoplasmic ratio with only a thin rim of cytoplasm (Figures. 1C, D). The nucleus consists of mostly uncondensed chromatin with few smaller clumps of condensed chromatin (Figure. 1C). When animals were macerated into a single cell suspension after a 30 min BrdU pulse, only cells with a neoblast morphology were labelled (n = 198) (Figure. 1D). On ultrastructural level, all cells that incorporated BrdU were small in size and possessed a thin rim of cytoplasm (Figures. 1E). These data suggest that neoblasts were the only dividing cells.

We next addressed the distribution of somatic stem cells in adults. BrdU labelling and ultrastructural analyses revealed a solely parenchymal distribution of S-phase cells (Figures. 1F-I). The majority of stem cells were located along the lateral sides of the animal, fewer cells were present also closer to the midline (Figures. 1F, G). Anterior to the statocyst, proliferating cells were almost completely absent. Notably, proliferating cells were never found in the epidermis of BrdU labelled animals (n = 300+) (Figures. 1F-I). These observations were further confirmed by ultrastructural investigations (Figure. 1I). Our data indicate that all epidermal cells were exclusively renewed from parenchymally located neoblasts.

We further followed the differentiation potential of BrdU labelled stem cells (Figures. 1J-L) in *I. pulchra*. BrdU pulse-chase experiments (Figures. 1K, L) revealed the differentiation of neoblasts into various cell types after a 10 days chase period (Figures. 1J-L). As mentioned above, all BrdU labelled cells exhibited a stem cell phenotype after 30 min BrdU exposure. After 10 days chasing time however, only 6.5% of labelled cells possessed stem cell morphology (11 out of 167) while 93.5% possessed a differentiated cell phenotype (156 out of 167).

### *Ipiwi1 *expression in adults, during regeneration and during development

**Figure 2:**
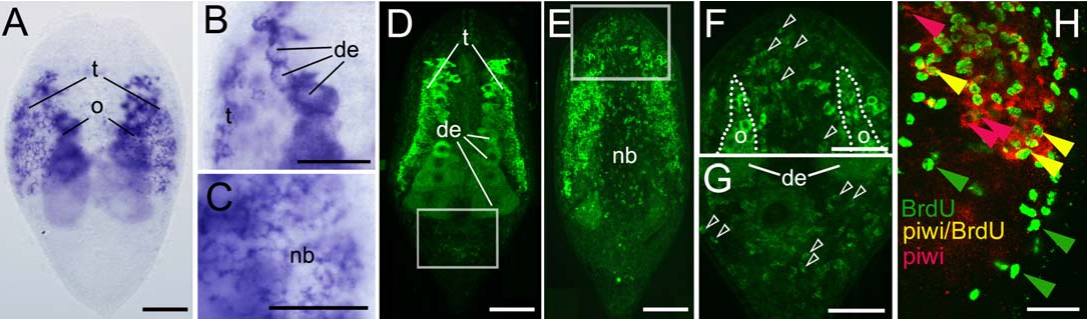
***Ipiwi1 *mRNA expression (A-C) and protein localization (D-G) and BrdU/*ipiwi1 *(H) double labelling in *I. pulchra***. (A) Whole mount *ipiwi1 in situ *hybridization of an adult specimen. (B) Detail of developing eggs (de) and testes (t). (C) Dorsal focal plane showing *ipiwi1 *mRNA expressed in neoblasts (open arrowheads). (D, E) Confocal projections of Ipiwi1 protein localisation in testes and developing eggs (D) and in neoblasts (nb) (E). (F) Detail of the anterior region of (E) demonstrating Ipiwi1 positive cells (open arrowheads). (G) Detail of the posterior region of (D) demonstrating Ipiwi1 positive cells (open arrowheads). (H) Double staining of stem cells in S-phase (green) and *ipiwi1 *positive cells (red). Confocal projection (1,02 μm) shows the presence of BrdU-only labelled cells (green arrows), *ipiwi1*-only labelled cells (red arrows) as well as BrdU/*piwi *double labelled stem cells (yellow arrows). In all figures, anterior is to the top. Scale bars (A, D, E) 100 μm; (B, C, F-H) 50 μm.

Next, we analyzed the expression dynamics of *piwi-*like genes in *Isodiametra pulchra *in adults, during development, regeneration, starvation and after irradiation. From *I. pulchra*, we have isolated two *piwi*-like genes, *ipiwi1 *and *ipiwi2 *(Additional files 1 and 2, Figures. S1, S2), both comprising the conserved Piwi and Paz domains (Additional file 3, Figure. S3), which are characteristic for members of the Piwi/Ago family [46,47]. Comparable to most organisms studied so far, *ipiwi2 *appeared to have a germ line specific expression (Additional file 4, Figures. S4A-C). Interestingly, *ipiwi1 *showed in addition to the germ line, an expression pattern extended to somatic stem cells (Figure. 2), a situation only known from rhabditophoran flatworms, sponges and cnidarians [33,36,37,43-45]. Therefore we focussed in this study on *ipiwi1*. To localize Ipiwi1 protein, we have generated a specific polyclonal antibody (Additional file 4, Figure. S4F).

In adult animals, *ipiwi1 *mRNA and protein were localized in a subpopulation of somatic stem cells and gonads (Figure. 2) while the sense probe did not show any signal (Additional file 4, Figure. S4D). *Ipiwi1 *positive cells in testes comprised two bands on the lateral sides of the animal which consisted of spermatogonia and spermatocytes (Figures. 2A, B, D). All stages of female germ cells expressed *ipiwi1 *including oogonia, oocytes and mature eggs (Figures. 2A, B, D). We further localized *ipiwi1 *mRNA expression in neoblasts in the region posterior to the statocyst but not in the posterior end of the animal (Figures. 2A, C). In contrast, few Ipiwi1 protein positive cells were also found anterior to the statocyst (Figures. 2E, F) and in the tail region (Figure. 2G). These data suggest that Ipiwi1 protein functions also in differentiating neoblasts, a situation similar to triclad flatworms [33,34,37]. Double labelling of *ipiwi1 *with BrdU revealed *ipiwi1*-only labelled cells, BrdU-only labelled cells as well as *ipiwi1*/BrdU double labelled stem cells (Figure. 2H). These data suggest that *ipiwi1 *was restricted to only a subpopulation of neoblasts.

**Figure 3:**
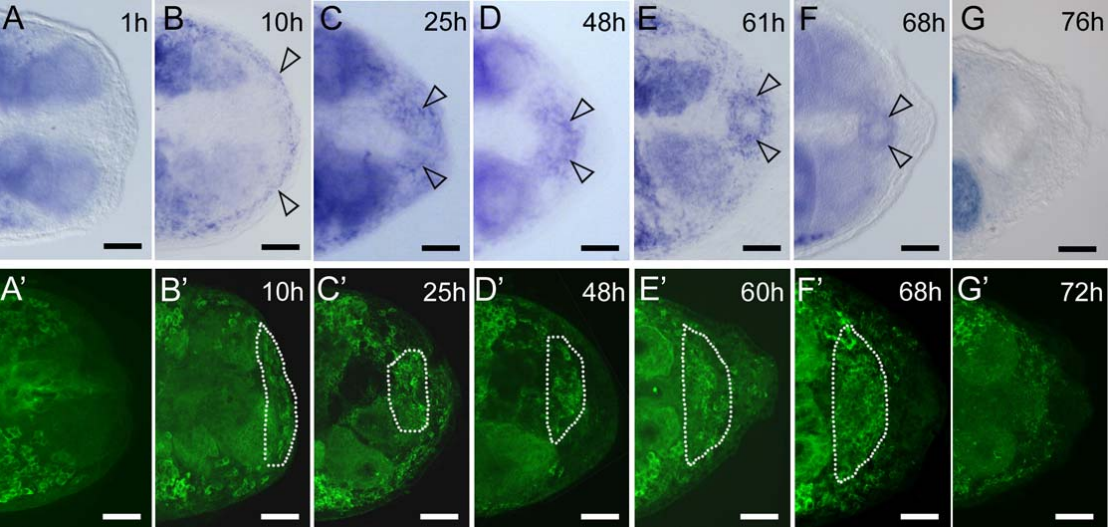
***Ipiwi1 *mRNA expression (A-G) and protein localization (A'-G') during posterior regeneration**. One hour after cutting (A, A'), *ipiwi1 *expression could not be detected at the regeneration site. After 10 hours, *ipiwi1 *was upregulated below the epidermis (arrows) (B, B'). At 25 hours postamputation (C, C') a significant proportion of cells within the regeneration blastema were *ipiwi1 *positive. From 48 hours onwards *ipiwi1 *expression and protein were present in the differentiating genital blastema (open arrows in D-F'). (G, G') After 76 h, *ipiwi1 *expression reached default levels. Scale bars 50 μm.

The process of regeneration in acoel flatworms was earlier examined on both morphological and immunohistochemical level but no molecular analyses have been performed to date [23,24,26,30,48,49]. Here we show *ipiwi1 *expression dynamics during successive stages of tail regeneration (Figure. 3, Additional file 5, Figure. S5) (this species is not able to perform anterior regeneration). One hour after initial amputation, *ipiwi1 *could not be detected at the regeneration site (Figures. 3A, A'). 10 hours postamputation however, a small rim of *ipiwi1 *positive cells became visible below the epidermis (Figures. 3B, B'). At 25 hours after amputation, *ipiwi1 *was upregulated within the small blastema (Figures. 3C, C'). From 48 to 68 hours of regeneration, *ipiwi1 *expression was detected in neoblasts that were organized in a ring-shaped structure (Figures. 3D-F') and outlining the subsequent developing reproductive organs. *Ipiwi1 *expression was upregulated only locally within the regeneration blastema but not in anterior regions of the animals (Additional file 5, Figure. S5). As regeneration proceeded, blastemal cell differentiation was paralleled by a gradual decrease in *ipiwi1 *expression (Figures. 3F-G').

**Figure 4:**
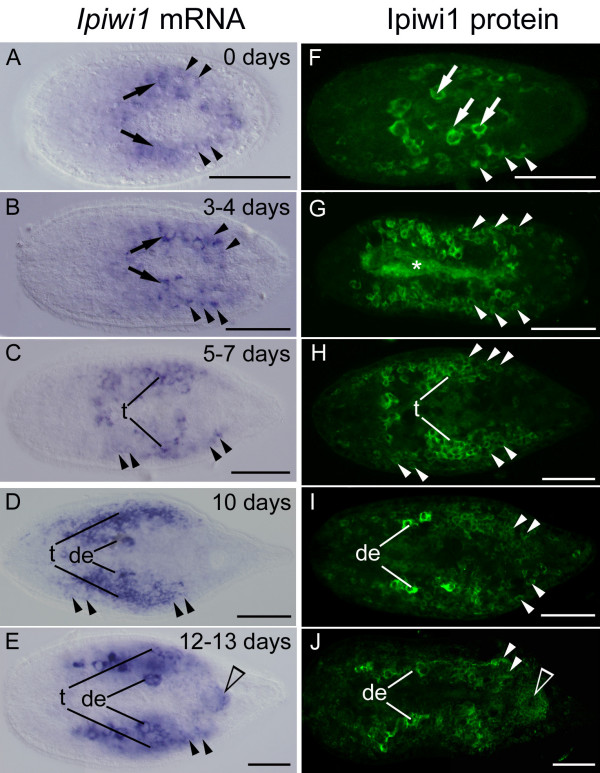
***Ipiwi1 *expression (A-E) and Ipiwi1 protein (F-J) during postembryonic development of *I. pulchra***. In freshly hatched animals, a subset of somatic neoblasts was visible as small *piwi *expressing cells (arrowheads) beside six to eight larger strongly stained primordial germ cells (that also express nanos, see text) (arrows in A, B, F). Until day seven neoblast number increased, PGCs multiplied and gave rise to testes and ovaries. At day seven testes and developing eggs could be observed (C, H). At days 10 and 12, a chain of developing eggs was present medially and testes were present along the lateral margin (D, E, I, J). Note the accumulation of *ipiwi1 *in the genital blastema (open arrowhead in E, J) which gives rise to the genital organs. A similar genital blastema was observed during regeneration (see Figure. 4). In all pictures, anterior is to the left. t, testes; de, developing eggs; (asterisk) autofluorescence of digested diatoms in the central syncytium. Scale bars 100 μm.

We next examined the expression of *ipiwi1 *throughout different stages of postembryonic development (Figure. 4). In freshly hatched *I. pulchra*, small parenchymally located somatic neoblasts and several larger *ipiwi1 *positive primordial germ cells were present in the central region of the animal (Figures. 4A, F). Those larger *ipiwi1 *positive cells gave rise to testes and ovaries and we confirmed the nature of these cells by double-labelling with an *I. pulchra *specific *nanos *probe (De Mulder, unpublished). The presence of primordial germ cells in freshly hatched *I. pulchra *suggested an embryonic segregation of the germ line in this species. The number of *ipiwi1 *positive cells increased up to four days post hatching (Figures. 4B, G) and distinct *ipiwi1 *stained testes were present after one week (Figures. 4C, H). Chains of developing eggs could be discerned after 10 days of postembryonic development (Figures. 4D, I). The number of *ipiwi1 *expressing somatic stem cells gradually increased during postembryonic development. A ring shaped structure of *ipiwi1 *positive cells accounted for the genital blastema (Figure. 4E), a structure identical to the differentiating genital blastema after 42 hours to 68 hours of regeneration (compare with Figure. 3G). The critical role of neoblasts became apparent by treatment with *ipiwi1 *dsRNA during development. The functional knock-down of *ipiwi1 *in developing worms resulted in a lethal phenotype (see below).

### Manipulation of the acoel stem cell system by hydroxyurea, radiation, and starvation

**Figure 5:**
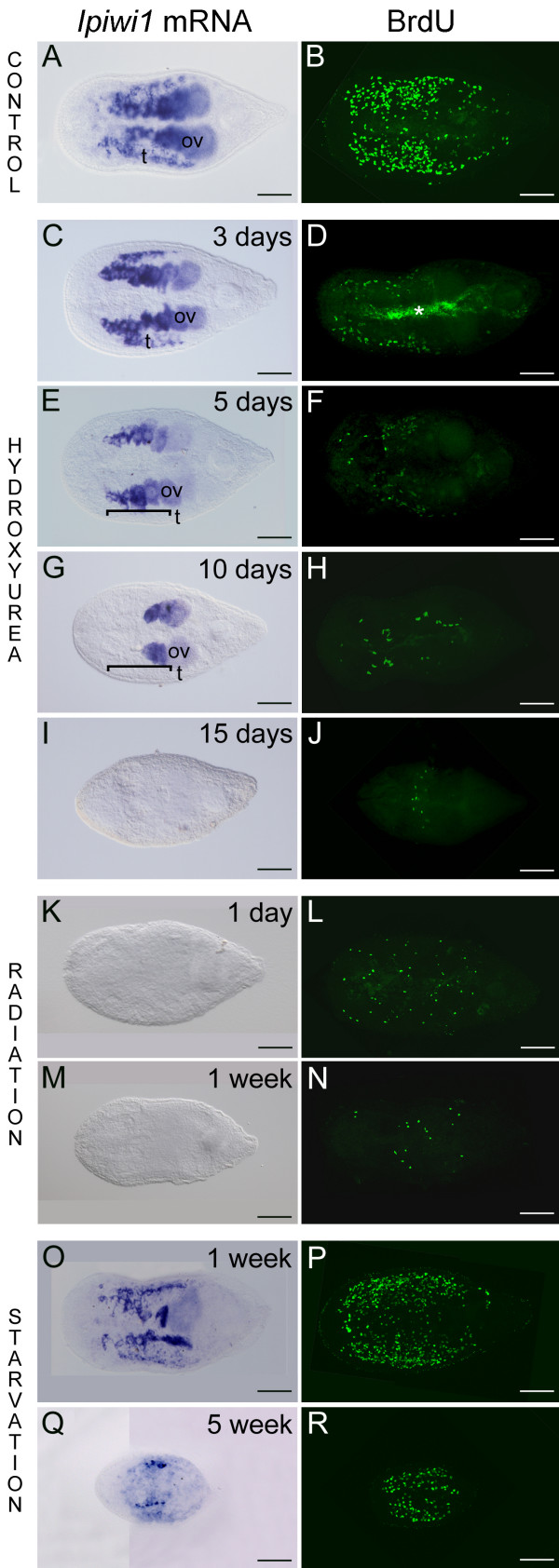
***Ipiwi1 *mRNA expression and cell proliferation (BrdU) in controls (A, B), during hydroxyurea treatment (C-J), after irradiation (K-N), and during starvation (O-R)**. (C) Upon three days HU treatment *ipiwi1 *mRNA could not be detected in neoblasts but was still present in testes (t) and ovaries (ov). (E) At five days of HU treatment *ipiwi1 *mRNA was present in all cells of the ovaries (ov) but not in testes (t, bracket indicates region of testes). (G) After 10 days *ipiwi1 *mRNA remained only in mature eggs; (t, testes; bracket indicates region of testes) (G). After 15 days no *ipiwi1 *mRNA could be detected (I) The number of S-phase cells strongly decreased during hydroxyurea treatment (D, F, H) and only single S-phase cells were found after 15 days (J). Irradiation (K-N) resulted in a complete elimination of *ipiwi1 *expression (K, M) and a strong reduction in the number of S-phase cells after one day (L) and one week (N) post irradiation. During starvation (O-R), *ipiwi1 *expression and protein localization were weakly reduced after one week (O, P). After five weeks of food deprivation, dramatic degrowth led to a strong reduction of *Ipiwi1 *mRNA and BrdU (Q, R). In all figures, anterior is to the left. t, testes; o, ovaries. Asterisk marks autofluorescence of diatoms within the gut. Scale bars 100 μm.

The inhibition of DNA-synthesis by hydroxyurea (HU) leads to an arrest of proliferating cells in the S-phase of the cell cycle and a pause of cell cycle progression [50] by inhibition of the ribonucleotide reductase [51]. We have applied hydroxyurea treatment for 18 days to halt the cell proliferation of stem cells and germ cells. After three to five days of HU treatment *ipiwi1 *expression of neoblasts was abolished and the number of somatic S-phase cells was drastically reduced (Figures. 5A-F). After 10 days, *ipiwi1 *expression persisted only in mature eggs and no *ipiwi1 *expression could be detected in the region of the testes (Figures. 5G, H). These results indicated that germ cell proliferation was interrupted but differentiation of oogonia was still possible. Moreover, the faster cell turnover in the testes resulted in an earlier reduction of *ipiwi1 *expressing cells (Figures. 5E, F). After 15 days of HU treatment *ipiwi1 *expression and cell proliferation of somatic stem cells were completely eradicated (Figures. 5I, J). The decrease in cell proliferation in the ovaries became apparent by the reduction in the production of eggs. Controls produced the following average number of eggs per animal per day: 1.07 at the start of the experiment, 0.99 after three days, 1.009 after five days, 1.45 after 10 days, 1.45 after 15 days (n = 287). In the HU treatment group egg numbers decreased from 1.09 at the start of the experiment to 0.79 after three days, 0.31 after five days, 0.017 after 10 days, and no eggs were laid anymore after 15 days HU treatment. These data demonstrate that we can use HU to manipulate and study stem cell- and germ cell development in *I. pulchra*.

Radiation is a widely used method in flatworm research to selectively destroy the stem cell system, which in turn stops maintenance of physiological homeostasis, cell renewal and regenerative capability [10,52-54]. In order to study the effect of irradiation on stem cell gene expression in acoels, we performed irradiation experiments with *I. pulchra*. We found that *ipiwi1 *expression was completely abolished one and seven days after irradiation, while the expression of the housekeeping gene *ipefα *(*Isodiametra pulchra *elongation factor alpha) persisted (Figures. 5K, M and Additional file 4, Figures. S4G-I). Furthermore, neoblast proliferation was drastically reduced one day and one week after irradiation (Figures. 5L, N). These results confirmed that in *I. pulchra *neoblasts can be eliminated by irradiation. Notably, few cells were still detectable by BrdU incorporation at one day (Figure. 5L) and one week (Figure. 5N) postradiation. It is possible that certain cells conduct intensified DNA repair which could lead to the incorporation of BrdU [55]. Another possibility is that certain stem cells were in a less radiosensitive phase of the cell cycle during radiation and started to divide and to incorporate BrdU. However, our results suggest that neither DNA repair nor the presence of radio resistant stem cells were able to reconstitute the entire stem cell population since irradiation led to death of the animals.

To date, nothing is known of the effect of starvation on the stem cell system of acoels. For this reason, we examined the expression dynamics of *ipiwi1 *during starvation in *I. pulchra *(Figures. 5O-R). After prolonged starvation the number of *ipiwi1 *positive cells was diminished, animals were drastically reduced in size and completely devoid of reproductive organs on morphological level. In *I. pulchra*, small *ipiwi1 *positive germ cells remained even after several weeks of starvation (Figures. 5Q, R). After refeeding, animals regrew again to adult stage within one month. These results suggest that degrowth of the animals, the reduction of reproductive organs, and the plasticity of the stem cell system during starvation is a feature how *I. pulchra *deals with food deprivation.

### *Ipiwi1 *RNA interference in adults, during regeneration and during development

In order to examine the function of *piwi*-like genes in *Isodiametra pulchra*, we applied RNA interference in adults, during development and regeneration. We examined the effect of the loss of *ipiwi1 *mRNA and protein by whole mount *in situ *hybridization of *ipiwi1*, the expression of the *vasa*-like gene *ipvasa*, by Ipiwi1 protein localization, and by BrdU labelling after 7 and 21 days of *ipiwi1 *dsRNA application. We confirmed the specificity of *ipiwi1 *and *ipiwi2 *dsRNA probes for silencing their respective target (Additional file 6, Figure. S6).

**Figure 6:**
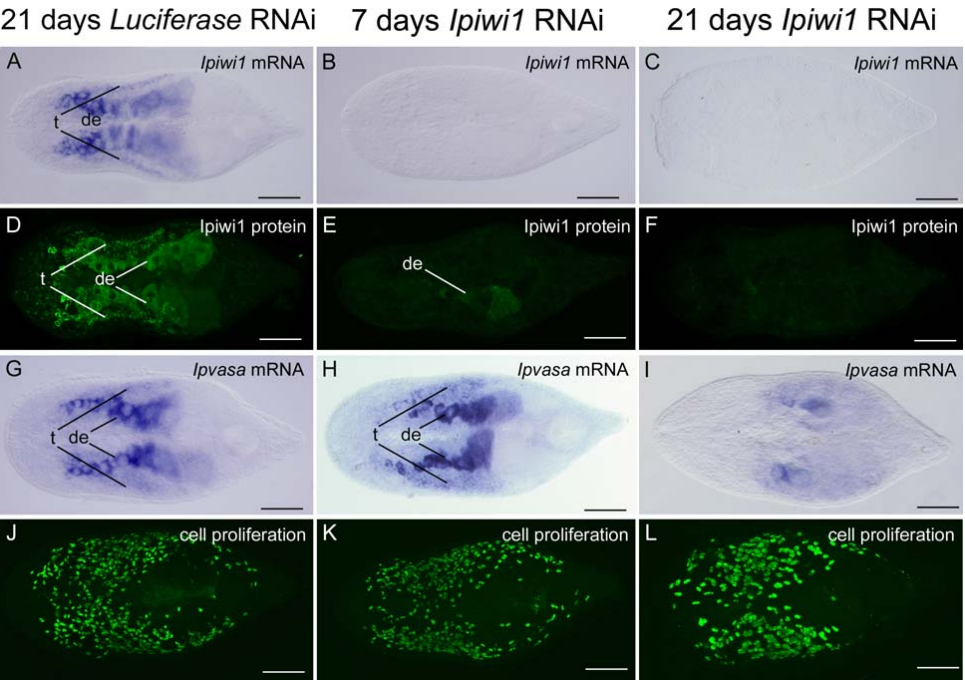
**Influence of *Ipiwi1 *RNAi on adult *I. pulchra *at seven and 21 days of *ipiwi1 *dsRNA treatment**. RNAi with *luciferase *control dsRNA did not show any effect on the level of i *piwi1 *expression (A), Ipiwi1 protein (D), *ipvasa *expression (G) or cell proliferation (J). *Ipiwi1 *mRNA was abrogated after seven days or 21 days of RNAi (B, C). Ipiwi1 protein was strongly reduced after seven days of *ipiwi1 *RNAi (E) and was completely eliminated after 21 days of *ipiwi1 *RNAi (F). *Ipvasa *expression was still prominent after seven days of *ipiwi1 *RNAi (H) and became significantly reduced after 21 days of *ipiwi1 *dsRNA treatment (I). Cell proliferation remained high up to 21 days of *ipiwi1 *dsRNA treatment (K, L). The specimen in figure 7L was processed for *in situ *hybridization before immunocytochemistry and therefore the nuclei appear larger. This protocol however did not alter cell number. In all figures, anterior is to the left. (t) testes; (de), developing eggs. Scale bars 100 μm.

In adults, *luciferase *dsRNA was applied as control and no noticeable mock effects were observed regarding *ipiwi1 *expression, BrdU incorporation and animal morphology (Figures. 6A, D, G, J). In contrast, *ipiwi1 *RNAi treatment led to an elimination of *ipiwi1 *mRNA and protein after seven and 21 days (Figures. 6B, C, E, F). *Ipiwi1 *RNAi resulted in a subsequent reduction in *ipvasa *expression after three weeks of treatment (Figures. 6H, I). Remarkably, *ipiwi1 *knock-down had at that time no apparent effect on stem cell proliferation and the phenotype of the animals (Figures. 6K, L).

A comparable role of *ipiwi1 *was observed during regeneration (Additional file 7, Figure. S7). Animals were cut twice - at one and two weeks of *ipiwi1 *RNAi treatment respectively - and were analyzed after 21 days, i.e. seven days after the final amputation. *Ipiwi1 *dsRNA treated regenerates lacked *Ipiwi1 *mRNA and protein (Additional file 7, Figures. S7B, D), had reduced *ipvasa *expression (Additional file 7, Figures. S7E, F), but preserved normal cell proliferation (Additional file 7, Figures. S7K, L), and were able to rebuild the missing body parts. However, these animals were unable to produce viable offspring. Taken together, these results suggest that *Ipiwi1 *is not involved - fulfils a redundant function - in the regulation of stem cell maintenance in adult and regenerating animals, but is crucial for offspring development.

**Figure 7:**
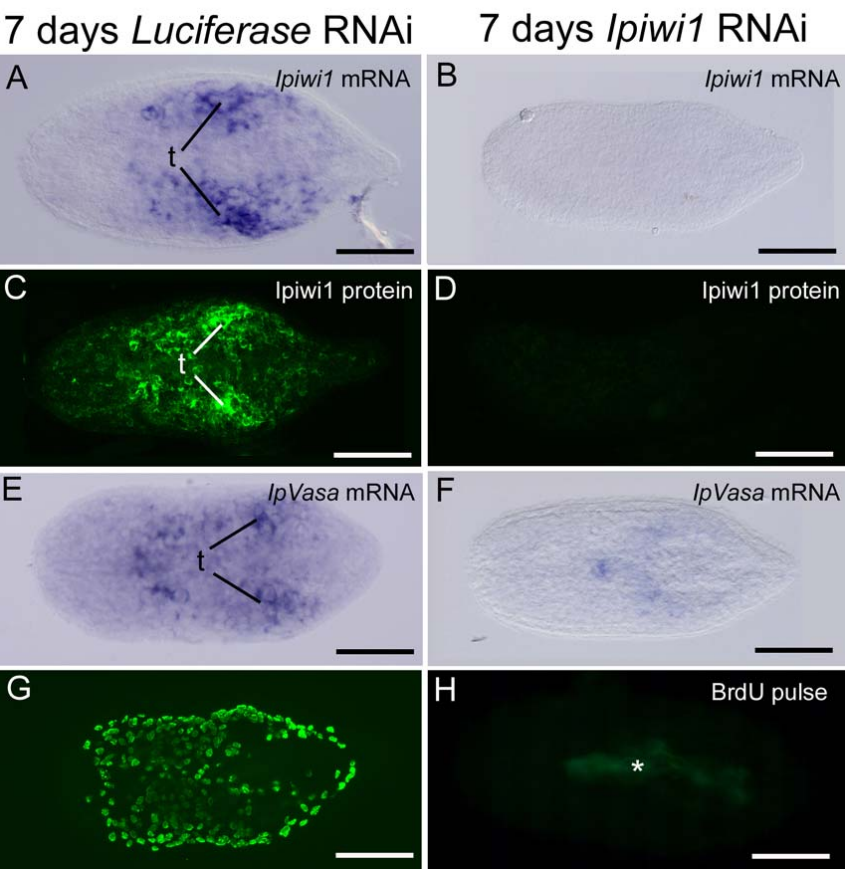
**Effect of *ipiwi1 *RNAi on development of *I. pulchra *after seven days of *ipiwi1 *dsRNA treatment**. As a control, RNAi with *luciferase *dsRNA was performed which did not lead to any change in *ipiwi1 *or *ipvasa *mRNA expression (A, E), Ipiwi1 protein (C) or cell proliferation (G). After seven days of *ipiwi1 *RNAi treatment, *ipiwi1 *and *ipvasa *mRNA and protein were drastically reduced (B, D, F) and cell proliferation had completely stopped (H). All *ipiwi1 *knock-down juveniles died before eight days of postembryonic development. In all figures, anterior is to the left. Autofluorescence of diatoms is marked with an asterisk. (t) testes. Scale bars 100 μm.

Finally, we wanted to address whether *ipiwi1 *had an essential function during development of *I. pulchra*. Therefore we eliminated *ipiwi1 *already in developing eggs of adult worms to abolish maternal *ipiwi1 *mRNA. As such, the term development used here includes all stages from a maturating egg within an adult, to embryonic and postembryonic stages. Eggs from adult worms, which were treated with *ipiwi1 *dsRNA for two weeks died without hatching. Embryos collected from one week *ipiwi1 *dsRNA treated adults hatched, but had abrogated *ipiwi1 *mRNA (Figure. 7B) and Ipiwi1 protein (Figure. 7D). They also did not retain *ipvasa *expression (Figure. 7F), completely lacked proliferating cells (Figure. 7H), and juveniles died within the first week of postembryonic development. These data suggest that *ipiwi1 *has an essential function during development.

## Discussion

### Acoels possess a potent stem cell system that is responsible for development, homeostasis, growth and regeneration

In recent years it has been shown that flatworms can serve as suitable model systems for understanding basis mechanisms of stem cell biology, regeneration, and aging [2,56-59]. Here we characterized the stem cell system of the acoel *Isodiametra pulchra *and clearly illustrated that *I. pulchra *possesses neoblast-like proliferating cells, earlier also described for the acoels *Convolutriloba longifissura *and *Convoluta naikaiensis *[29,30]. Epidermal cells as well as all other cell types from the three germ layers were exclusively renewed from these mesodermally located stem cells. A similar mode of tissue homeostasis and epidermal replacement is known from rhabditophoran Platyhelminthes such as macrostomids [2,60-63], triclads [5,6,52,64-67] and neodermata [68-73]. Within the Bilateria, a stem cell population crucial for development, tissue homeostasis and regeneration is hitherto only known from Acoela and Rhabditophora. In the cnidarian *Hydra*, I-cells serve as stem cells for most tissues, whereas two epithelial cell lineages guarantee for epithelial tissue homeostasis [74]. Likewise, in other taxa with high regeneration capacity such as sponges [45], several stem cell populations ensure tissue specific homeostasis.

In basal metazoan taxa with high regeneration and transdifferentiation capacity such as sponges and cnidarians, *piwi*-like genes play a role in the regulation of gonadal and somatic stem cells [43,45]. Notably, studies on the expression of *piwi*-like genes of key positioned taxa such as catenulids, nemertodermatids, gnathostomulids, gastrotrichs are lacking. Here we showed that in the adult *I. pulchra ipiwi1 *is expressed in a subpopulation of somatic stem cells and in germ cells. Regarding the crucial phylogenetic position of acoels, our data give evidence that *piwi *expression extended to somatic stem cells might have persisted from basal Bilateria to higher organisms including ascidians and human blood cells [75,76].

Since *I. pulchra *is not able to regenerate a new head, we focussed in the current study on posterior regeneration. During the first days, *ipiwi1 *expression was locally upregulated underneath the wound epithelia. As regeneration proceeded, differentiation of the tissue was paralleled by decrease of *piwi *expression. Notably there was an apparent similarity between *piwi *expression dynamics during formation of the genital organs during development and regeneration. Such a local *piwi *upregulation was also found during regeneration in triclads [34], as well as during regeneration and development in *Macrostomum lignano *[77].

During the development of animals with sexual reproduction, a biological decision has to be made to separate soma (body cells) from the germline (gametes). However, in some phyla, such as sponges, cnidarians, acoels and rhabditophoran flatworms, the border between those two lineages is not clearly made and germ cells can be formed *de novo *from somatic stem cells (reviewed in [78]). Here we show that in the acoel *I. pulchra*, germ cell precursors are already present in freshly hatched worms, suggesting an embryonic formation of the germline. Although in flatworms it was initially supposed that the germline is formed postembryonically [79,80], several publications recently showed the presence of germ cells in late embryos or freshly hatched worms [3,77,81]. However, despite the fact that germ cells might be already present in late embryos of *I. pulchra *and some rhabditophoran flatworms they maintain somatic neoblasts during adulthood which retain the capacity to differentiate into germ cells [82,83].

### *Ipiwi1 *expression dynamics following stem cell depletion by HU treatment, irradiation and starvation

In *I. pulchra*, prolonged HU treatment resulted in a drastic decline in stem cell proliferation and *ipiwi1 *expression. The faster elimination of *ipiwi1 *expression and BrdU in somatic stem cells and testes, compared to ovaries could be explained by the faster cell turnover in these tissues [84]. Notably, after 10 days of HU treatment, few cells were still able to incorporate BrdU. These cells might be gonadal cells or slow cycling neoblasts, activated upon stem cell depletion [60]. In triclad flatworms hydroxyurea was applied to detect fast and slow cycling neoblasts [85]. In the parasitic platyhelminth *Schistosoma mansoni *it was found that both sexes were sensitive to hydroxyurea treatment [86]. Interestingly, it was shown that hydroxyurea had no effect on metamorphosis of miracidia [87]. In the cnidarian *Hydra *HU was used to reduce the number of interstitial cells [88] and to follow nerve cell and nematocyte differentiation [89]. To conclude, our data demonstrate that we can use HU to manipulate and study stem cell- and germ cell development in *I. pulchra*.

Since neoblasts are the only proliferating cells in rhabditophoran flatworms, radiation is a commonly used method to confirm stem cell specific gene expression [4,53,84,90-92]. In this study, we showed that a similar situation was observed after depleting the stem cell population of acoels by radiation. Radiation drastically reduced the expression of *ipiwi1*, confirming his stem cell specific expression. One week post radiation, few cells were still able to incorporate BrdU. Further experiments will reveal if these cells are activated slow cycling neoblasts or gonadal stem cells which were shown to possess higher radio tolerance in rhabditophoran flatworms [84].

Food deprivation resulted in degrowth of *I. pulchra*. During prolonged starvation, animals successively decreased in body size, possessed reduced gonads, and showed a diminished proliferation activity. After refeeding however, animals regrew again to adult size. Comparably, some annelids [93], nemerteans [94] and rhabditophoran flatworms [6] are able to starve for months and undergo degrowth during that period. The terrestrial triclad *Arthurdendyus triangulatus *undergoes natural periods of growth and degrowth correlated with the availability of its prey - the earthworm [95-97]. Upon starvation, adult animals resorb their tissues and deplete body reserves [98] and cannot be distinguished from juvenile animals [99]. The striking cellular responses of freshwater triclads to degrowth include the reduction of cell proliferation, a decrease in cell numbers, and autophagy [100-103]. Similar observations of growth and degrowth were found in the macrostomid flatworm *M. lignano *[84,104]. We conclude that degrowth, and the reduction of reproductive organs are features how *I. pulchra *deals with food deprivation.

### *Ipiwi1 *function is essential for acoel development

In order to analyse the function of *piwi-*like genes in acoels, we established a non-invasive RNAi protocol by soaking. *Ipiwi1 *RNA interference during development resulted in a lethal phenotype, demonstrating the crucial role of *ipiwi1 *during development. Although *ipiwi1 *is expressed in a subpopulation of somatic stem cells and in germ cells no visible phenotype could be observed after prolonged RNAi treatment regarding homeostasis and regeneration. The absence of a clear phenotype could be explained by the fact that other *piwi-*like genes might compensate for *Ipiwi1 *function. At the moment, we cannot exclude this possibility since the genome of *I. pulchra *is not yet available and screening with several different degenerated primers did not result in the isolation of additional *piwi*-like genes. We can exclude a redundancy with *ipiwi2 *since *ipiwi1/ipiwi2 *double RNAi did not lead to a more severe phenotype (data not shown).

Although redundant *piwi-*like genes might exist in *I. pulchra*, it is intriguing that redundancy would act during homeostasis and regeneration, but not during development. These observations indicate that stem cells might be differentially regulated and expression of different *piwi-*like genes might vary during development and homeostasis [77]. Further characterization of all *piwi-*like genes might clarify if we deal with different stem cell populations or if stem cells are differentially regulated.

## Conclusions

In this study, we presented the acoel *Isodiametra pulchra *as suitable model organism to address developmental questions in this understudied phylum. We established stable laboratory cultures of *I. pulchra *with unlimited availability of offspring the whole year through, and developed a whole mount ISH protocol and a simplified RNAi method by soaking.

Summarizing all data we can conclude that (1) acoel neoblasts are the only proliferating cells in *Isodiametra pulchra*, (2) acoel stem cells show a characteristic morphology on the light and electron microscopical level, (3) neoblasts are exclusively located parenchymally with a lack of proliferating cells in the epidermis, (4) cell renewal for tissue homeostasis, during growth and regeneration is based exclusively on parenchymal stem cells, (5) *piwi *expression in *I. pulchra *is, in addition to the germline, present in a subpopulation of somatic neoblasts, (6) *I. pulchra *exhibits a high plasticity upon starvation accompanied by substantial degrowth and the reduction of reproductive organs. Refeeding leads to a full restoration of size and reproduction, (7) irradiation leads to the elimination of neoblasts and finally to the death of the animals, (8) functional knock-down of *Ipiwi1 *reveals an essential role of *Ipiwi1 *during development.

## Methods

### Animal culture

*Isodiametra pulchra *(Acoela, Acoelomorpha) was kept in petri dishes with nutrient-enriched f/2 artificial sea water [105] and fed ad libitum with diatoms (*Nitzschia curvilineata*). Climate chamber conditions were 20°C and 60% humidity with 14/10 hours day/night cycle.

### Cloning of *piwi*-like genes and sequence analysis

Partial Sequences of *Ipiwi1 *and *Ipiwi2 *were obtained from an EST project (Ladurner and Agata, unpublished). Concatenation of five EST's resulted in the full length ORF of *Ipiwi1 *(accession number *Ipiwi1 *[EMBL:AM942741]); while another clone represented a partial sequence of *Ipiwi2*. Full length sequence of *Ipiwi2 *was obtained by 5'RACE-PCR using a SMART RACE cDNA amplification kit (BD Bioscience) with the sequence specific primers 5'-GAATTGGCTCATGCGGGTCAGTC-3' and 5'-GGAAGTCCTCCCGCATCTTGTCC-3'. The revealed PCR product was cloned using a pGEM-T vector system I (Promega) and sequenced by MWG (Germany). Nested primers were made in the newly obtained sequence: 5'-CTCGAACTTCAGCAACCGCATGA-3', 5'-GTTCTGGCATGGAAGG-GGATTGG-3' and 5'-GGGAGGGCTGAAATCGACATGGTA-3' and used for nested PCR with the *I. pulchra *cDNA phage library as template. The obtained PCR product was cloned into a pCR II-TOPO vector (Invitrogen) and sequenced by GATC (Konstanz, Germany). The accession number of *Ipiwi2 *is [EMBL:AM942742].

### Whole mount in situ hybridization

Whole mount in situ hybridization was carried out as described previously for *M. lignano *(Pfister et al. 2007), except for the proteinase K treatment (7 min for *I. pulchra*). Riboprobes were generated using the DIG RNA labelling KIT SP6/T7 (Roche), following the manufacturers protocol.

Template DNA for producing DIG-labelled probe was made by standard PCR (primer couple for *Ipiwi1*: 5'-CATGCTGGAGATGGGCAAGATCAC-3' and 5'-GGTGCCGGAGATTTCATTGCTCTC-3; for *Ipiwi2*: 5'-GCATGAGCCAATTCATC-AGTCGAG-3' and 5'-GGCAGCTCACCGTCATTCATCTCT-3'; for *IpVasa*: 5'-ACCCACGAAGGCATCAACTTC-3' and 5'-TCGCATCTCTTCTTCATCTCG-3' [EMBL_FN298396]); for *IpEfα *5'-GTCAGTATTGTCGTCATTGGCC-3' and 5-'GCTCCATTCTTTAAACCAGGGC-3' ([EMBL_FN298397]) which produced ISH probes for *Ipiwi1 *(826 bp), *Ipiwi2 *(865 bp), *Ipvasa *(882 bp) and *IpEfα *(624 bp). During hybridization riboprobes were used at working concentrations of 0,05 for *Ipiwi1 *and *Ipvasa *and 0,1 ng/μl for *Ipiwi2 *and *IpEfα*, respectively. Pictures were made using a Leica DM5000 microscope and a Pixera Penguin 600CL digital camera.

### Immunohistochemistry

Antibody stainings were performed as previously described (Ladurner et al., 2005) with the following modifications: animals were fixed for only 30 min with 4% PFA at room temperature (RT). Multiple PBS-T (0,1%) washes (3 × 5 min, 1 h at RT) were followed by 30 min blocking in PBS-BSA-T (1%) (RT). Primary antibody was incubated overnight in PBS-BSA-T (4°C) (1/1000 for Ipiwi1). After washing with PBS-T (0,1%) (3 × 5 min), specimen were incubated in secondary antibody (1/200 FITC-swine-anti-rabbit, 1 h RT, DAKO) and washed again 3 × 5 min in PBS-T. Specimen were mounted with Vectashield (VECTAR) and analyzed with a Leica DM5000. Confocal images were made with a Zeiss LSM 510.

To localize Ipiwi1 proteins, we have generated a specific polyclonal antibody (Additional file 4, Figures. S4). Primary polyclonal Ipiwi1 antibody was produced by GenScript (GenScript Corp, NJ, USA). The following peptide was used for immunisation: DREERPRFINDENV(C) (aa 98-111).

### Electron microscopy and immunogold labelling were performed according to Bode et al.[60]

Double labelling of S-phase cells (BrdU) and *Ipiwi1 *expressing cells (in situ hybridization) Preceding fixation, animals were pulsed for 30 min with 5 mM BrdU to label neoblasts in S-phase [61]. In situ hybridization was performed as described above, except for color development, which was carried out with Fast Red, in order to obtain fluorescent staining (Sigma, F4648). After *in situ *hybridization, animals were rinsed in ddH_2_O and further processed through the BrdU staining protocol [61] except for protease XIV treatment, which was done at a final concentration of 0,1 mg/ml for 20 minutes at 37°C.

### Single cell maceration

In order to prevent algae contamination, animals were starved for 2 days. For each maceration, 3 adult animals were BrdU pulsed for 30 min (5 mM in F/2), washed twice with culture medium and directly further processed (BrdU pulse) or left for 10 days under standard culture conditions in the dark (BrdU pulse-chase). Specimens were gradually relaxed for 5 min in 7,14% MgCl_2 _and dissociated in CMF/1% trypsin solution for 1 hour at 37°C. During maceration, animals/cells were carefully mixed every 15 minutes. Cells were pelleted, supernatant was removed, and cells were resuspended in 200 μl PFA (4% in PBS) and fixed for 40 min at room temperature. Cells were transferred on coated slides (DAKO, S2024), and dried for 10 minutes. 6 × 5 min PBS-T (0,1%) washing steps were performed, followed by 45 min incubation in 2 N HCl (37°C). After 3 × 5 min PBS-T washes, unspecific staining was blocked during 30 min, in PBS-BSA (1%)-Triton (0,1%). Primary antibody was used in a final concentration of 1/800 in PBS-BSA-T (mouse anti BrdU, Roche) and incubated overnight at 4°C. The next day, cells were washed 3 × 5 min in PBS-T and incubated for 1 hour in secondary antibody (goat anti mouse FITC; 1/200, DAKO). Excessive antibody was removed by 5 × 5 min incubation in PBS-T and cells were mounted in Vectashield. Pictures were taken using a Leica DM5000 microscope.

### Western blot

Animals were starved for 1 day. Total protein of 650 animals was extracted in 100 μl 2× Slab/100 μl PBS and loaded onto 12% acrylamide gels (90 min, 150 V). Protein was blotted on polyvinylidene fluoride membranes (90 min, 25 V) (Immobilon-P; Millipore) and blocked for 2 h with PBS (pH 7,4) containing 0.3% Tween 20, 1% skimmed milk powder. Blots were incubated overnight at 4°C in primary antibody with a final concentration of 1 μg/ml for Ipiwi1. After washing the blots for 3 × 10 min in PBS-Tween (0,3%), membranes were incubated with alkaline phosphatase-conjugated anti-mouse immunoglobulin (1/10,000 Sigma, 2 h, RT). Finally, after several washing steps (8 × 10 min), immunocomplexes were detected using nitro blue tetrazolium: 5-bromo-4- chloro-3 indolyl phosphate (LifeTechnology).

### Post embryonic development, regeneration, and starvation

About 1000 staged eggs were collected of *I. pulchra*. During the whole postembryonic development (19 days), 50 juveniles were fixed each day and stored in methanol until further processed for ISH and immunohistochemistry.

To obtain regenerating animals, 500 *I. pulchra *were cut at the tail region. Every day, 40 animals were fixed and stored in methanol (-20°C) until further processed for ISH and immunohistochemistry respectively.

During starvation, worms were kept in petri dishes filled with culture media (f/2) without food. Medium was changed twice a week. Every week, a batch of 50 animals was fixed and stored in MeOH until further processing.

### Hard X-ray irradiation

Intact worms were exposed to 60 Gray, using a linear Accelerator (8 MeV, 400 cGy/min; Radio-Oncology, Medical Hospital, Innsbruck). Animals were fixed one hour, one day, one week, two weeks and three weeks postirradiation and examined for *piwi *expression and BrdU incorporation.

### Hydroxyurea treatment

A batch of 400 adults (30 - 40 days old) was treated with 2,8 mM hydroxyurea, a specific inhibitor of DNA synthesis (HU, Sigma H-8627) [106]. During the whole treatment (18 days), animals were kept continuously in the dark and HU medium was changed daily. Every second day, a batch of worms was pulsed for 30 min with BrdU (5 mM in F/2), relaxed and fixed for in situ hybridisation, as described earlier.

### RNA interference

An RNA interference protocol by soaking was newly developed for *I. pulchra *using a dsRNA probe generated by an *in vitro *transcription system (T7 RibomaxTM Express RNAi System, Promega). The dsRNA probe used for RNAi overlaps completely with the ISH probes for *ipiwi1 *(bp 1304 - bp 2131) (Additional file 1, Figure. S1) and *ipiwi2 *(865 bp) (Additional file 1, Figure. S2). As a negative control for RNA interference, a 1002 bp Luciferase fragment was used (pGEM-luc Vector (Promega). dsRNA was diluted in f/2 culture medium to a final concentration of 3 ng/μl and supernatant was changed every 12 hours. Throughout the whole experiment, animals were fed ad libitum in 24 well plates (25 animals per well). Specimens were examined for BrdU incorporation, *piwi *mRNA and protein expression as well as the influence of *piwi *RNAi on *vasa *expression after 7 days and 21 days treatment. Survival, reproducibility and regeneration capacity were followed during the whole experiment (d = 21).

## Competing interests

The authors declare to have no competing financial or other interest in relation to their work.

## Authors' contributions

KDM contributed to conception and design of the project, contributed to acquisition of all data, analysed and interpreted the data and was involved in drafting the manuscript. DP, AKG, and MH significantly contributed to ISH establishment and GK initially participated in *piwi *isolation. MW and BE contributed in regeneration experiments. WS and MT participated in transmission electron microscopy and sectioning. GB contributed in *piwi *results and manuscript drafting. PL has designed the study, was involved in the radiation experiments, interpreted results, and helped to draft the manuscript. All authors read and approved the final manuscript.

## Supplementary Material

Additional file 1 **Figure S1: Nucleotide sequence and predicted protein product of *Ipiwi1***. Conserved PAZ and PIWI domains highlighted in blue (PAZ) and green (PIWI). The piwi box within the piwi domain is marked in red. Start and stop codon are underlined and marked in bold. ISH primers are underlined within the sequence. Accession number for *Ipiwi1 *(AM942741). Click here for file

Additional file 2 **Figure S2: Nucleotide sequence and predicted protein product of *Ipiwi2***. Conserved PAZ and PIWI domains are highlighted in blue (PAZ) and green (PIWI). The piwi box within the piwi domain is marked in red. Start and stop codon are underlined and marked in bold. ISH primers are underlined within the sequence. Accession number for *Ipiwi2 *(AM942742). Click here for file

Additional file 3 **Figure S3:. Alignment of predicted *piwi*-like genes from *I. pulchra *with *piwi*-like genes from other species**. (A) Amino acid alignment of the conserved PAZ domain. (B) Amino acid alignment of the conserved PIWI domain. The PIWI box is highlighted in purple. Amino acids indicated with green asterisks are supposed to create a binding pocket for the 5'phosphate group of binding RNA. Red asterisks indicate putative RNase active site carboxylate residues. Amino acids indicated in purple can distinguish members of the piwi and argonaute subfamily. The Genbank accession numbers: *Isodiametra pulchra Ipiwi1 *(AM942741); *Isodiametra pulchra Ipiwi2 *(AM942742); *Macrostomum lignano Macpiwi *(AM942740); *Schmidtea mediterranea Smedwi1 *(DQ186985) *Smedwi2 *(DQ186986); *Dugesia japonica DjPiwi1 *(AJ865376); *Podocoryne carnea Cniwi *(AAS01181); *Caenorhabditis elegans PRG1 *(NP492121); *Drosophila melanogaster DmPiwi *(AF104354); *Strongylocentrotus purpuratus Seawi *(AY014899); *Homo sapiens Hiwi *(AF104260). Click here for file

Additional file 4 **Figure S4: *Ipiwi2 *expression, *Ipiwi1 *and *Ipiwi2 *control sense probes, *Ipiwi1 *Western Blot and radiation controls**. *Ipiwi2 *whole mount in situ hybridization (A) with detail of expression in testes (t) (B) and in developing eggs (de) (C). (D) *Ipiwi1 *sense control. (E) *Ipiwi2 *sense control. (F) Western blot of Ipiwi1 polyclonal antibody, showing a signal at the expected size (100 kDa). (G-I) Hard X ray radiation of 60 Gray did not result in a significant downregulation of the housekeeping gene *Isodiametra pulchra *elongation factor alpha (*IpEfα*). *IpEfα *Control (G); *IpEfα *expression after one day (H) and one week (I) postirradiation. Scale bars 100 μm in (A, D, E, G, H, I), 50 μm in (B) and 25 μm in (C). Click here for file

Additional file 5 **Figure S5: **. Overview of *Ipiwi1 *expression and dynamics during posterior regeneration in *Isodiametra pulchra*. This whole mount overview clearly demonstrates that *Ipiwi1 *is only locally upregulated within the regeneration blastema (for details see Figure. 4). Expression of *Ipiwi1 *mRNA (A-I) and protein localization (J-R). One hour after cutting (A; J) and up to five hours postamputation (B, K) *Ipiwi1 *could not be detected at the regeneration site by in situ hybridization. *Ipiwi1 *was upregulated in the regeneration blastema (arrow) after 10 hours (C, L) and 25 hours postamputation (D, M). From 42 hours onwards *Ipiwi1 *remained downregulated in the regeneration blastema (E-R) and *Ipiwi1 *expression and protein were only present in the differentiating genital blastema (open arrows in E-Q). Scale bars 100 μm. Click here for file

Additional file 6 **Figure S6: **Specificity of the dsRNA silencing of *Ipiwi1 *and *Ipiwi2*. RNA interference of *Ipiwi1 *resulted in the elimination of *Ipiwi1 *mRNA within 7 days (A) but *ipiwi2 *remained present (C). Likewise, RNA interference using *Ipiwi2 *dsRNA resulted in the elimination of *ipiwi*2 transcripts (D) but *ipiwi1 *was not affected (B). Scale bars 100 μm. Click here for file

Additional file 7 **Figure S7:. Effect of *Ipiwi1 *RNAi on the regeneration of *I. pulchra *after 21 days of *Ipiwi1 *dsRNA treatment**. RNAi with *luciferase *dsRNA did not show any effect on *Ipiwi1 *or *Ipvasa *mRNA expression (A, E), Ipiwi1 protein (C) or cell proliferation (G). After 21 days of *Ipiwi1 *dsRNA treatment *ipiwi1 *expression was eliminated both on mRNA (B) as well as on protein level and only weak *ipvasa *mRNA could be detected in remnant eggs (F). Notably, cell proliferation was not affected up to 21 days of *ipiwi1 *dsRNA treatment (H). (de) developing eggs; (t) testes. Scale bars 100 μm. Click here for file
