# Supplementary figures and images for: Characterization of the stem cell system of the acoel Isodiametra pulchra
Source: BMC Dev Biol. 2009 Dec 18;9:69. doi: 10.1186/1471-213X-9-69 (PMC2806412; doi:10.1186/1471-213X-9-69)

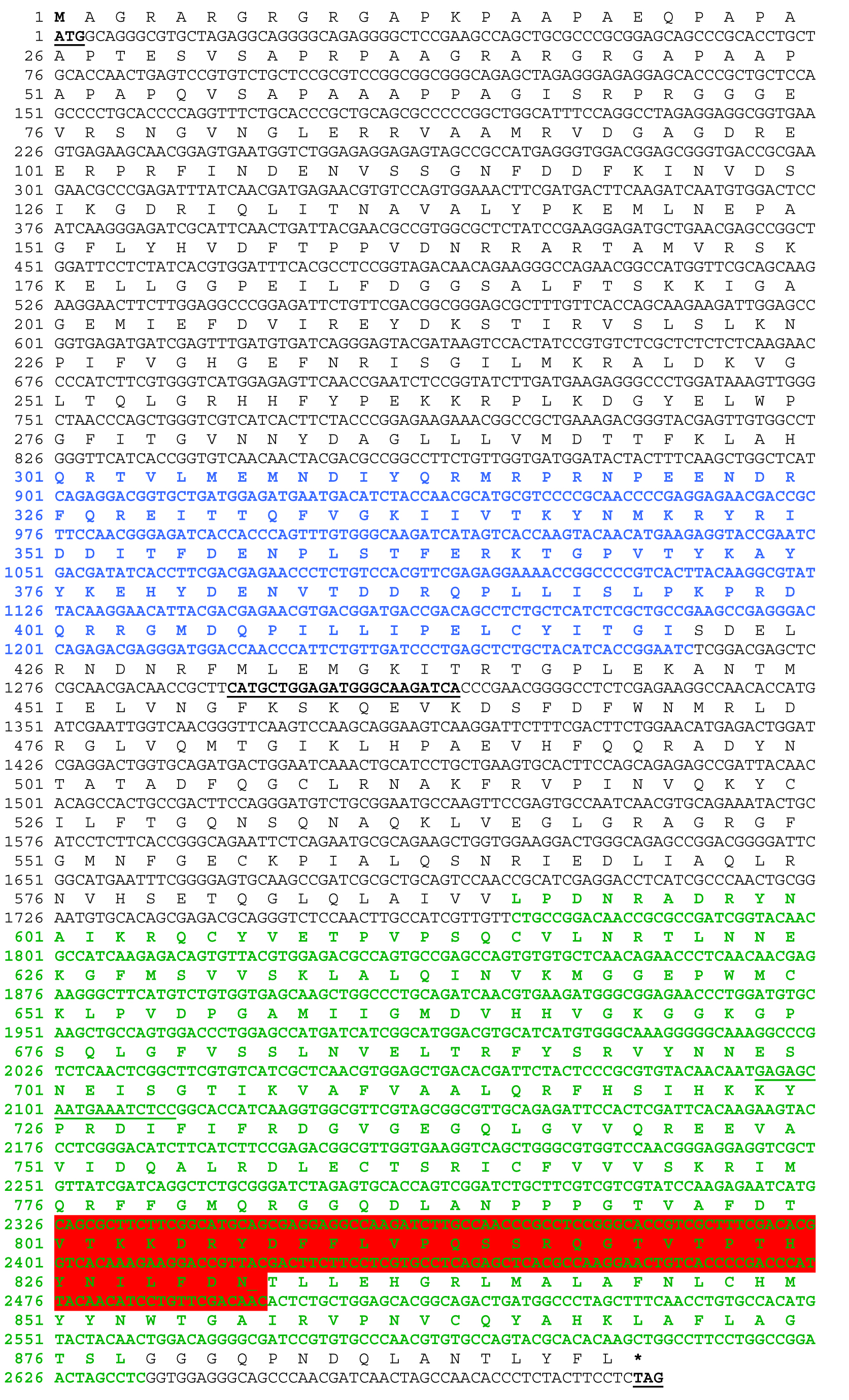

Supplement: Additional file 1 — Figure S1: Nucleotide sequence and predicted protein product of Ipiwi1. Conserved PAZ and PIWI domains highlighted in blue (PAZ) and green (PIWI). The piwi box within the piwi domain is marked in red. Start and stop codon are underlined and marked in bold. ISH primers are underlined within the sequence. Accession number for Ipiwi1 (AM942741). [file 1471-213X-9-69-S1.JPEG]

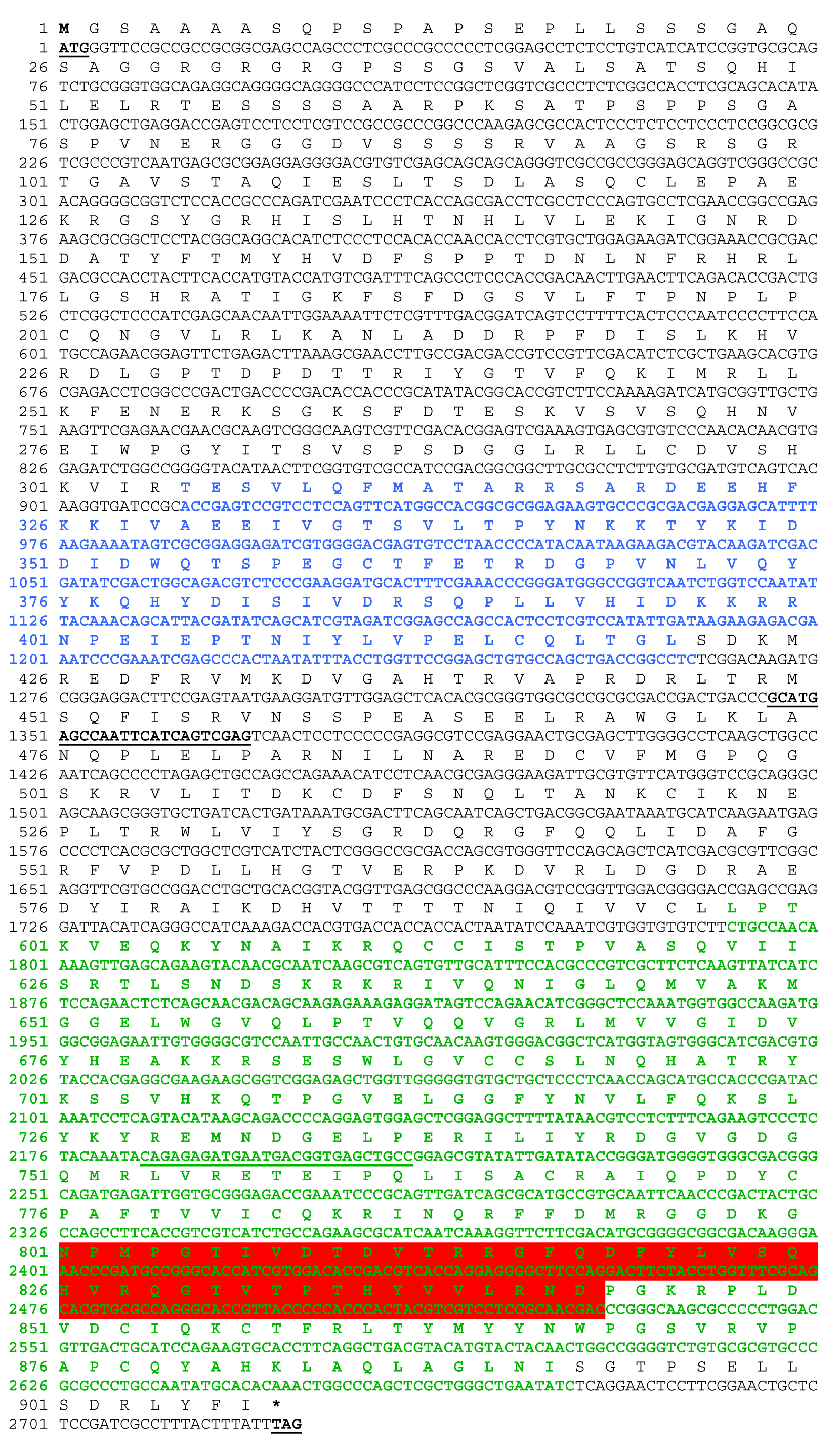

Supplement: Additional file 2 — Figure S2: Nucleotide sequence and predicted protein product of Ipiwi2. Conserved PAZ and PIWI domains are highlighted in blue (PAZ) and green (PIWI). The piwi box within the piwi domain is marked in red. Start and stop codon are underlined and marked in bold. ISH primers are underlined within the sequence. Accession number for Ipiwi2 (AM942742). [file 1471-213X-9-69-S2.JPEG]

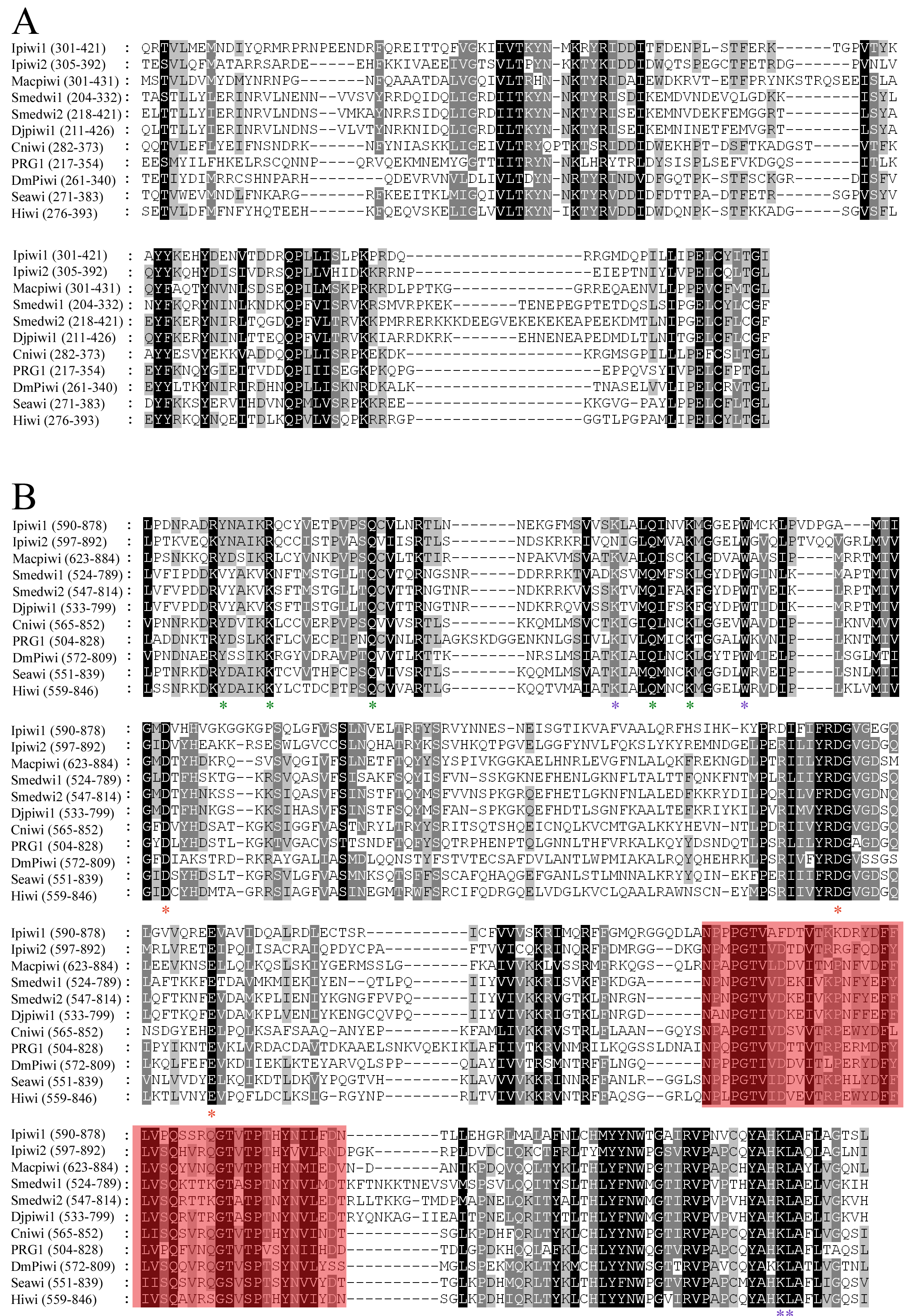

Supplement: Additional file 3 — Figure S3:. Alignment of predicted piwi-like genes from I. pulchra with piwi-like genes from other species. (A) Amino acid alignment of the conserved PAZ domain. (B) Amino acid alignment of the conserved PIWI domain. The PIWI box is highlighted in purple. Amino acids indicated with green asterisks are supposed to create a binding pocket for the 5'phosphate group of binding RNA. Red asterisks indicate putative RNase active site carboxylate residues. Amino acids indicated in purple can distinguish members of the piwi and argonaute subfamily. The Genbank accession numbers: Isodiametra pulchra Ipiwi1 (AM942741); Isodiametra pulchra Ipiwi2 (AM942742); Macrostomum lignano Macpiwi (AM942740); Schmidtea mediterranea Smedwi1 (DQ186985) Smedwi2 (DQ186986); Dugesia japonica DjPiwi1 (AJ865376); Podocoryne carnea Cniwi (AAS01181); Caenorhabditis elegans PRG1 (NP492121); Drosophila melanogaster DmPiwi (AF104354); Strongylocentrotus purpuratus Seawi (AY014899); Homo sapiens Hiwi (AF104260). [file 1471-213X-9-69-S3.JPEG]

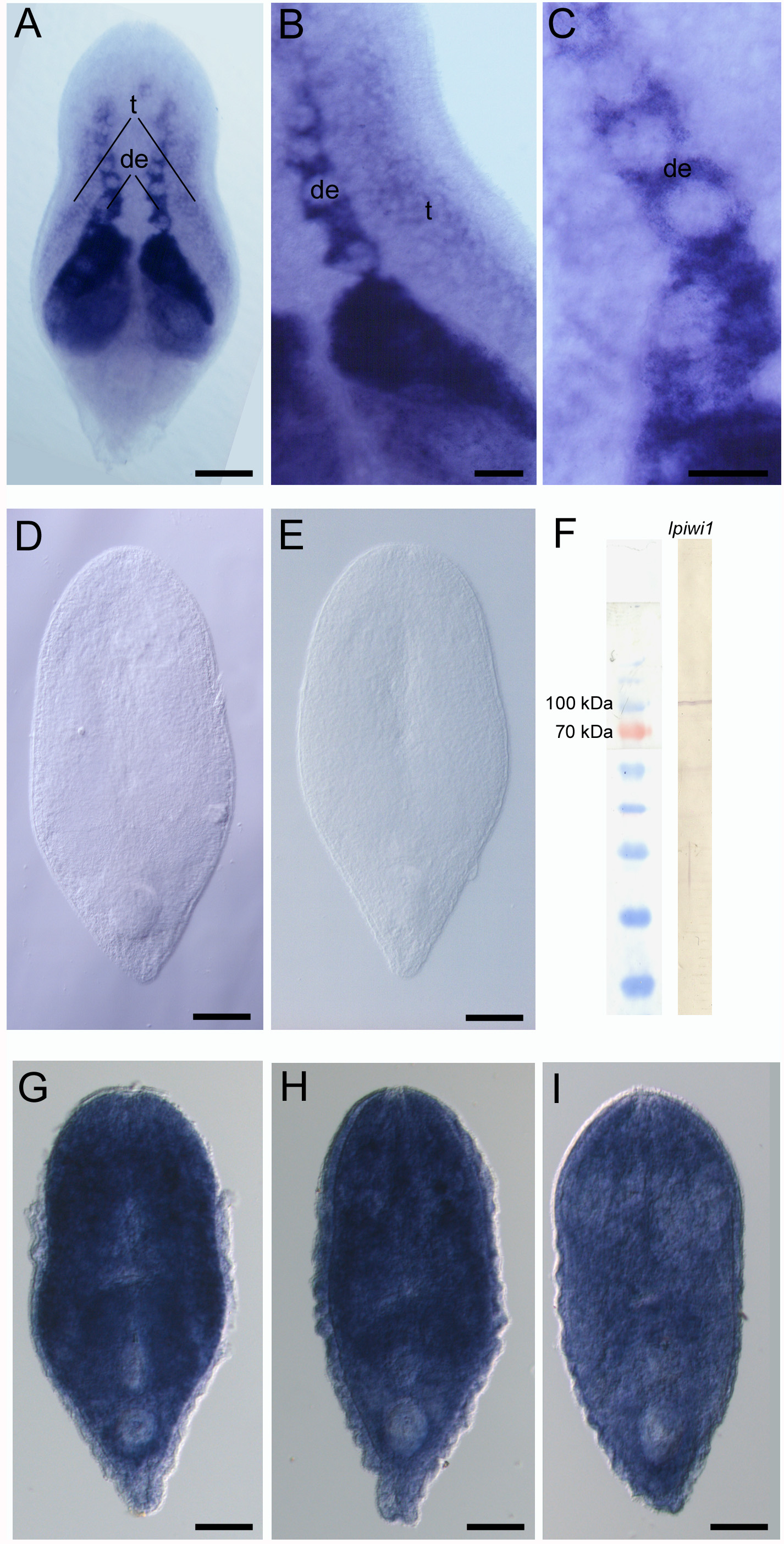

Supplement: Additional file 4 — Figure S4: Ipiwi2 expression, Ipiwi1 and Ipiwi2 control sense probes, Ipiwi1 Western Blot and radiation controls. Ipiwi2 whole mount in situ hybridization (A) with detail of expression in testes (t) (B) and in developing eggs (de) (C). (D) Ipiwi1 sense control. (E) Ipiwi2 sense control. (F) Western blot of Ipiwi1 polyclonal antibody, showing a signal at the expected size (100 kDa). (G-I) Hard X ray radiation of 60 Gray did not result in a significant downregulation of the housekeeping gene Isodiametra pulchra elongation factor alpha (IpEfα). IpEfα Control (G); IpEfα expression after one day (H) and one week (I) postirradiation. Scale bars 100 μm in (A, D, E, G, H, I), 50 μm in (B) and 25 μm in (C). [file 1471-213X-9-69-S4.JPEG]

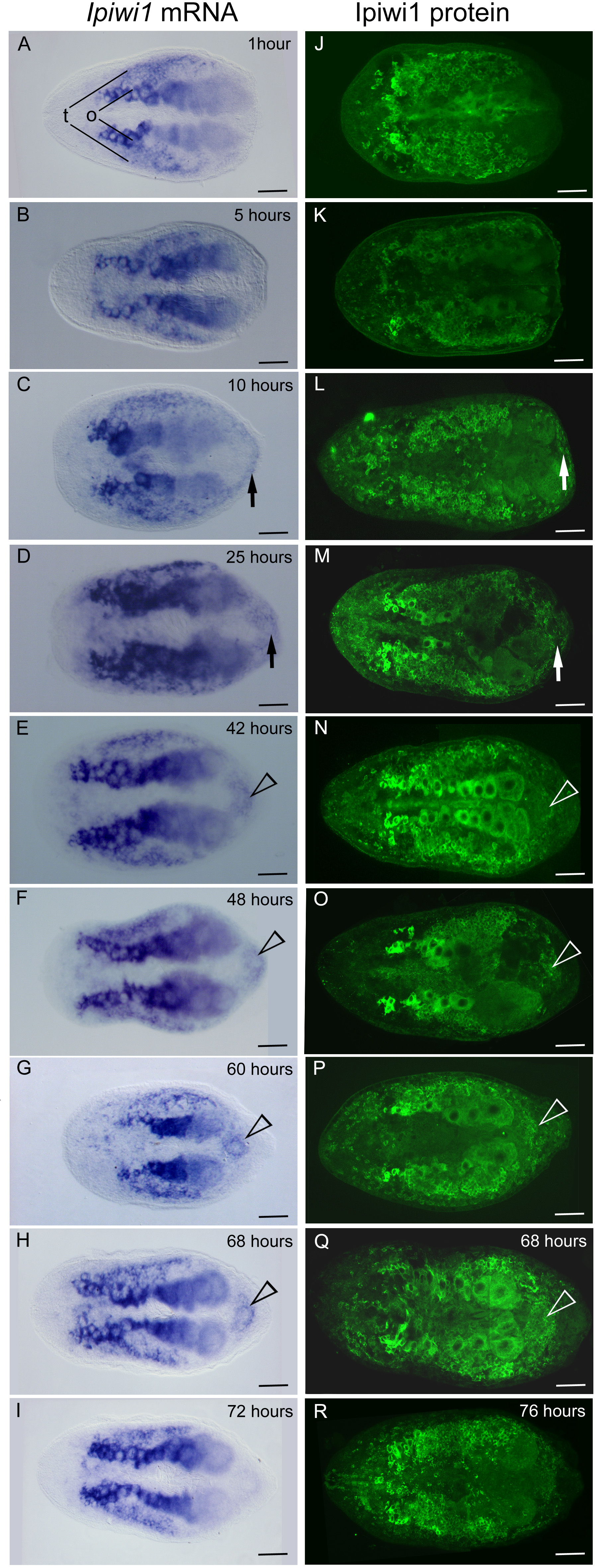

Supplement: Additional file 5 — Figure S5: . Overview of Ipiwi1 expression and dynamics during posterior regeneration in Isodiametra pulchra. This whole mount overview clearly demonstrates that Ipiwi1 is only locally upregulated within the regeneration blastema (for details see Figure. 4). Expression of Ipiwi1 mRNA (A-I) and protein localization (J-R). One hour after cutting (A; J) and up to five hours postamputation (B, K) Ipiwi1 could not be detected at the regeneration site by in situ hybridization. Ipiwi1 was upregulated in the regeneration blastema (arrow) after 10 hours (C, L) and 25 hours postamputation (D, M). From 42 hours onwards Ipiwi1 remained downregulated in the regeneration blastema (E-R) and Ipiwi1 expression and protein were only present in the differentiating genital blastema (open arrows in E-Q). Scale bars 100 μm. [file 1471-213X-9-69-S5.JPEG]

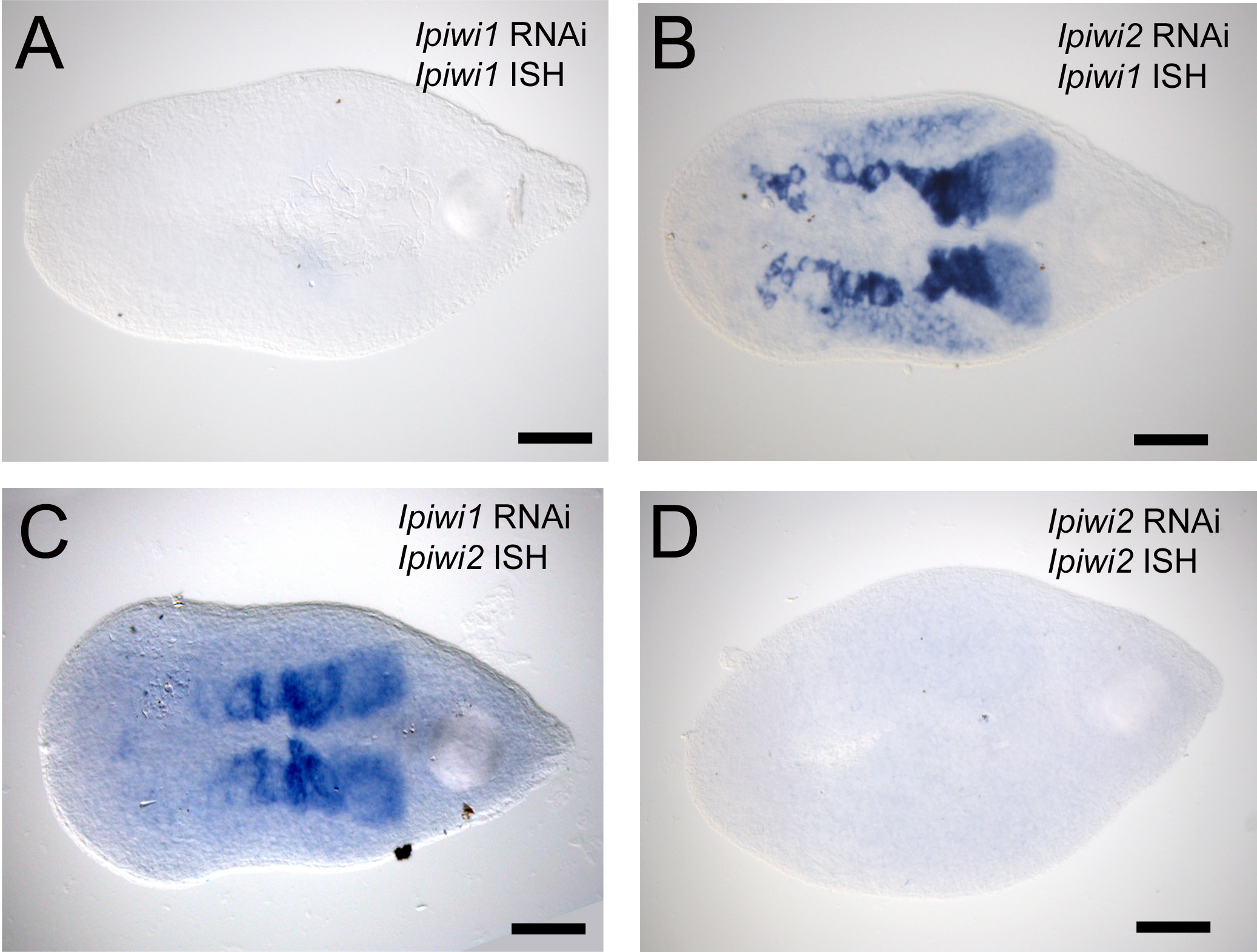

Supplement: Additional file 6 — Figure S6: Specificity of the dsRNA silencing of Ipiwi1 and Ipiwi2. RNA interference of Ipiwi1 resulted in the elimination of Ipiwi1 mRNA within 7 days (A) but ipiwi2 remained present (C). Likewise, RNA interference using Ipiwi2 dsRNA resulted in the elimination of ipiwi2 transcripts (D) but ipiwi1 was not affected (B). Scale bars 100 μm. [file 1471-213X-9-69-S6.JPEG]

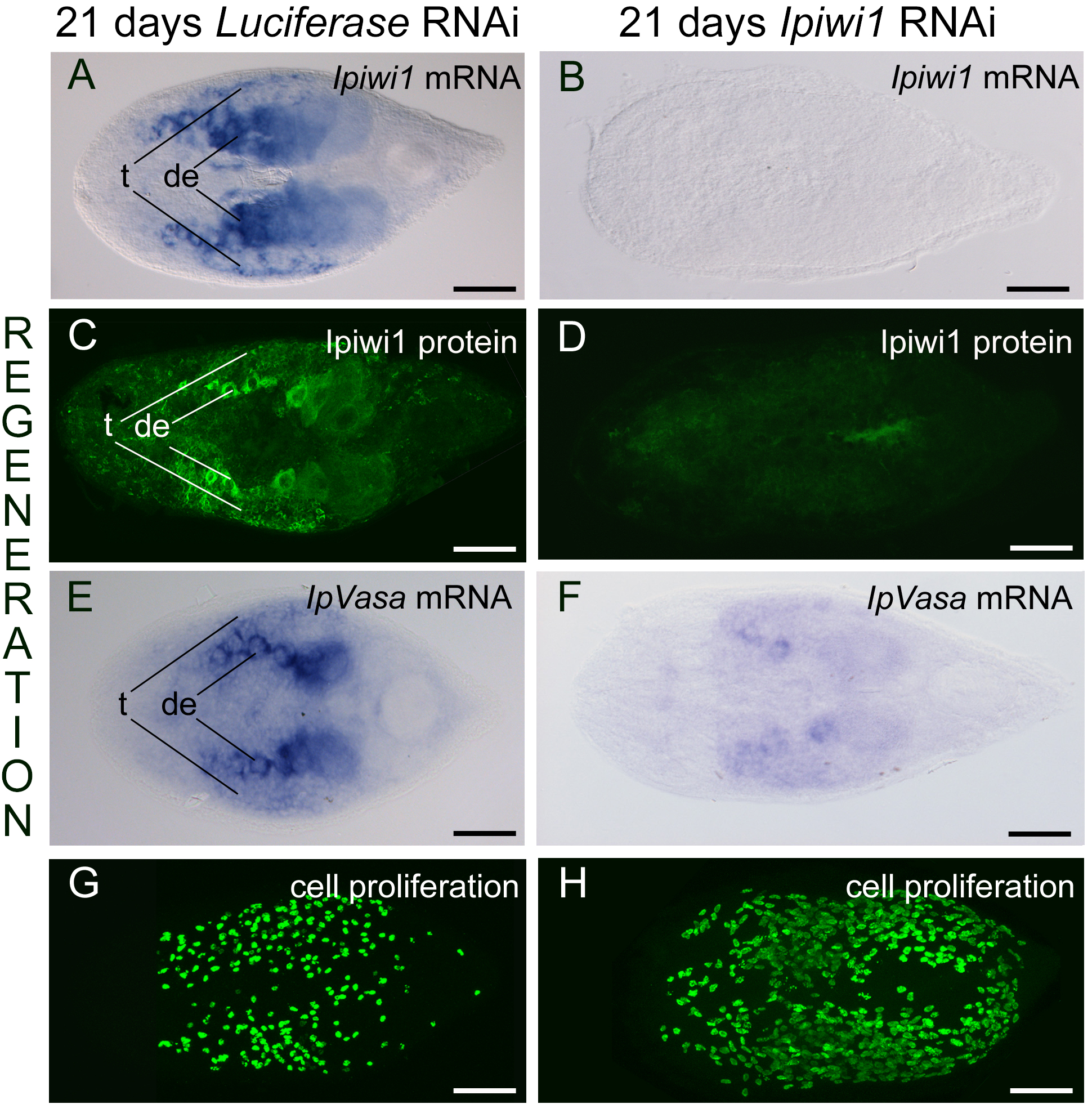

Supplement: Additional file 7 — Figure S7:. Effect of Ipiwi1 RNAi on the regeneration of I. pulchra after 21 days of Ipiwi1 dsRNA treatment. RNAi with luciferase dsRNA did not show any effect on Ipiwi1 or Ipvasa mRNA expression (A, E), Ipiwi1 protein (C) or cell proliferation (G). After 21 days of Ipiwi1 dsRNA treatment ipiwi1 expression was eliminated both on mRNA (B) as well as on protein level and only weak ipvasa mRNA could be detected in remnant eggs (F). Notably, cell proliferation was not affected up to 21 days of ipiwi1 dsRNA treatment (H). (de) developing eggs; (t) testes. Scale bars 100 μm. [file 1471-213X-9-69-S7.JPEG]
